# Compliance with hand disinfection in the surgical area of an orthopedic university clinic: results of an observational study

**DOI:** 10.1186/s13756-022-01058-2

**Published:** 2022-01-31

**Authors:** Claas Baier, Maren Tinne, Thomas von Lengerke, Frank Gossé, Ella Ebadi

**Affiliations:** 1 Institute for Medical Microbiology and Hospital Epidemiology, Hannover Medical School (MHH), Carl-Neuberg Str. 1, 30625 Hannover, Germany; 2 Department of Medical Psychology, Centre for Public Health and Healthcare, Hannover Medical School (MHH), Carl-Neuberg Str. 1, Hannover, Germany; 3 Department of Spinal Surgery and Conservative Orthopedics, Clinic of Orthopedics of Hannover Medical School (MHH) at DIAKOVERE Annastift, Anna-von-Borries-Str. 1-7, 30625 Hannover, Germany

**Keywords:** Hand hygiene, Compliance, Direct observation, Operation theatre, Surgery, Anesthesia

## Abstract

**Background:**

Hand hygiene using alcoholic hand rub solution is essential for the prevention of surgical site infections. There are several opportunities for hygienic hand disinfection (termed “hand hygiene” in the following) during immediate pre-, intra- and postoperative orthopedic patient care. However, the level of hand hygiene compliance among surgical and anesthesia staff in this context is unclear. Therefore, we conducted an observational study in operating theatres of an orthopedic university clinic in northern Germany during July and August 2020.

**Methods:**

One trained person directly and comprehensively observed hand hygiene compliance of surgical and anesthesia staff according to the WHO “My 5 moments for hand hygiene” model (WHO-5). In addition to cross-tabulations with Chi^2^ tests, multiple logistic regression models were used to study associations between occupational group, medical specialty, and compliance (both overall and for each WHO-5 indication). Models were adjusted for hand hygiene opportunities being associated with female or male healthcare workers, being located within or outside the operation room, and occurring in adult or pediatric surgery.

**Results:**

In total, 1145 hand hygiene opportunities during 16 surgeries were observed. The overall compliance was 40.8% (95% CI 37.9–43.6%), with a larger difference between surgical versus anesthesia staff (28.4% vs. 46.1%, *p* < 0.001) than between physicians versus nurses (38.5% vs. 42.9%, *p* = 0.13). Adjusting for sex, place of observation, and adult versus pediatric operation theatre, logistic regression analyses revealed a significant interaction between medical specialty and occupational group (*p* < 0.001). In particular, the odds for compliance were higher for anesthesiologists (47.9%) than for surgeons (19.6%) (OR = 4.8, 95% CI 3.0–7.6). In addition, compliance was higher in pediatric surgery (OR = 1.9, 95% CI 1.4–2.6). In general, WHO-5-stratified results were in line with these overall patterns.

**Conclusions:**

Hygienic hand disinfection compliance was approximately 41%. Notably, surgeons performed worse than anesthesiologists did. These results indicate that hand hygiene compliance in orthopedic surgery needs to be improved. Tailored interventions promise to be an appropriate way to address each occupational group’s specific needs.

**Supplementary Information:**

The online version contains supplementary material available at 10.1186/s13756-022-01058-2.

## Background

Hand hygiene (HH) using alcoholic hand rub solution is one of the most important measures to prevent nosocomial infections, including surgical site infections and transmission of pathogens (e.g., multidrug resistant bacteria) in hospitals, but implementation is not easy in practice [1, 2]. Compliance with surgical hand disinfection before putting on sterile gloves just prior to surgery is high and a routinized part of professional surgical practice [3]. However, there are also numerous opportunities for hygienic hand disinfection according to the WHO “My 5 moments for hand hygiene” model (WHO-5) in surgical facilities during immediate pre-, intra- and postoperative patient care. These indications may occur at several stages, for instance, during anesthesia induction, patient transport and positioning, or during immediate postoperative care. There are several reports showing that compliance with guidelines on hygienic hand disinfection is low in anesthesia and surgery settings [4–8]. Multitasking and a high workload [9] increase the risk for low compliance. Varying HH compliance across different occupational groups and medical specialties has been reported before [10]. Differences regarding job-specific workflows, knowledge and attitudes might explain this observation. Also, overestimation of one’s own compliance may play a role as it may cause impairment of actual compliance. For instance, in a survey of the physicians in our study clinic, self-reported HH compliance from 72% (“after contact with patient surroundings”) to 97% (“after body fluid exposure”) was found [11]. Notably, in that survey surgeons rated their own overall SSI-preventive compliance (including hand hygiene) higher than anesthesiologists [11].

Against this background, we aimed to assess HH compliance in the perioperative setting in this clinic by observation and to evaluate differences between occupational groups (nurses vs. physicians) and medical specialties (surgery vs. anesthesia).To our knowledge this is the first study scrutinizing the interaction of these two variables (occupational group and medical specialty) regarding HH compliance in the immediate pre-, intra- and postoperative elective orthopedic surgery setting. Compliance of surgical and anesthesia personnel was observed during entire operative procedures, i.e., from the arrival of patients in the surgical area to discharge from this area.

## Methods

A cross-sectional observational study was conducted in July and August 2020 in a specialized orthopedic hospital in Hannover, Germany. The hospital is a center for joint and knee arthroplasty as well as spine surgery. Another specialization of the hospital is pediatric orthopedic surgery. The vast majority of surgical procedures are elective and take place during regular working hours (8 am to 2 pm). In total, the clinic has six operation theatres, each with a separate anesthesia induction room. All rooms are located in a circumscribed and access-restricted surgical area of the hospital which also includes hallways and patient transfer zones. Overall, 57 physicians (45 surgeons and 12 anesthesiologists) and 33 nurses (23 surgical and 10 anesthesiological) worked in the surgical area during the study period (53 women and 37 men). Alcoholic hand rub solution is available throughout the surgical area in dispensers located near places of patient care activities (e.g., induction rooms, operation theatres). The stretcher trolleys and mobile operation tables are not equipped with dispensers. Following national guidelines in Germany and the study hospital’s internal guidelines, hand washing (with water and soap) is recommended primarily for removing visible contaminations (e.g., prior to begin of work or in rare cases of direct contamination of the hand with for instance with excretions), but nor for hygienic hand disinfection in terms of the WHO-5. For the present study, it was therefore decided to focus on hygienic hand disinfection with alcoholic hand rub solution. Surgical hand disinfection was not assessed.

One doctoral student (2nd first author/MT) was trained in direct observation according to WHO-5 (before patient contact, before an aseptic task, after body fluid exposure, after patient contact, and after contact with patient surroundings) at Hannover Medical School’s (MHH) Institute for Medical Microbiology and Hospital Epidemiology. Hygienic hand disinfection compliance was observed in the study hospital on 12 working days, with the observer following 16 patients continuously during the entire stay in the surgical area. The observer was instructed to accompany different teams in order to observe as many different healthcare workers as possible. For every HH opportunity, the parameters sex, occupational group and medical specialty of the healthcare worker, location, indication (WHO-5), and the context of adult versus pediatric surgery were documented (the latter parameter was operationalized by one of the six operation theatres being reserved to pediatric patients). The performance of the observer was validated at three different time points during the study period. For this, experienced infection control nurses from the study clinic supervised the observer. The concordance in compliance assessment was 92.6% across 81 observations. The study hospital’s staff was informed in advance regarding the hand hygiene compliance observation, and data collection was anonymous.

Statistical analyses were conducted with IBM® SPSS® Statistics 26 (IBM Corp., Armonk, NY, USA). Overall and stratified (i.e., subgroup-specific) compliance was calculated as percentages. In addition to cross-tabulations with Chi^2^ tests, we used binary logistic multiple regression models to study associations between occupational group and medical specialty on the one hand and compliance on the other hand (both overall and for each WHO-5 indication). The models were adjusted for opportunities associated with female or male healthcare workers, being located within or outside the operating room, and occurring in adult or pediatric surgery. The interaction term “occupational group*medical specialty” was integrated, and if significant, additional stratified regression models were conducted.

## Results

Table 1 gives an overview of HH observations and compliance overall and in different subgroups. In total, 1145 HH opportunities were observed, of which 190 occurred before patient contact (16.6%), 277 before an aseptic task (24.2%), 198 after body fluid exposure risk (17.3%), 221 after patient contact (19.3%), and 259 after contact with patient surroundings (22.6%). The overall compliance was 40.8% (95% CI 37.9–43.6%). Across the WHO-5 categories, the lowest compliance was observed after contact with patient surroundings (25.1%, 95% CI 20.1–30.7%), while compliance was highest after patient contact (61.1%, 95% CI 54.5–64.4%). Compliance as related to women only showed a tendency to be higher than for men (overall: 43.2% vs. 38.6%, *p* = 0.114).

**Table 1:** Results of hygienic hand disinfection compliance observation (n = 1145 opportunities observed)

Parameter	Compliant opportunities/observed opportunities (compliance in %)	By WHO-5				
		Before patient contact	Before aseptic task	After body fluid exposure risk	After patient contact	After contact with patient surroundings
All opportunities (O.)	467/1145 (40.8%)	55/190 (28.9%)	92/277 (35.0%)	120/198 (60.6%)	135/221 (61.1%)	65/259 (25.1%)
O. by women	235/544 (43.2%)	22/76 (28.0%)	46/133 (34.6%)	64/97 (66.0%)	66/92 (71.7%)	37/146 (25.3%)
O. by men	232/601 (38.6%)	33/114 (28.9%)	46/144 (31.9%)	56/101 (55.4%)	69/129 (53.5%)	28/113 (24.8%)
O. by physicians	215/558 (38.5%)	20/89 (22.5%)	52/142 (36.6%)	60/108 (55.6%)	58/115 (50.4%)	25/104 (24.0%)
O. by nurses	252/587 (42.9%)	35/101 (35.7%)	40/135 (29.6%)	60/90 (66.7%)	77/106 (72.6%)	40/155 (25.8%)
O. by surgical staff	97/342 (28.4%)	10/65 (15.4%)	6/27 (22.2%)	39/81 (48.1%)	24/67 (35.8%)	18/102 (17.6%)
O. by anesthesia staff	370/803 (46.1%)	45/125 (36.0%)	86/250 (34.4%)	81/117 (69.2%)	111/154 (72.1%)	47/157 (29.9%)
O. in operation theatres for adult surgery	324/867 (37.4%)	47/145 (32.4%)	64/217 (29.5%)	84/150 (56.0%)	91/161 (56.5%)	38/194 (19.6%)
O. in operation theatre for pediatric surgery	143/278 (51.4%)	8/45 (17.8%)	28/60 (46.7%)	36/48 (75.0%)	44/60 (73.3%)	27/65 (41.5%)
O. in anesthesia induction room (incl. patient arrival)	218/509 (42.8%)	34/119 (28.6%)	58/134 (43.3%)	50/66 (75.8%)	54/94 (57.4%)	22/96 (22.9%)
O. inside operation theatre	179/463 (38.7%)	14/54 (25.9%)	26/117 (22.2%)	56/105 (53.3%)	57/71 (80.3%)	26/116 (22.4%)
O. in postoperative care	70/173 (40.5%)	7/17 (41.2%)	8/26 (30.8%)	14/27 (51.9%)	24/56 (42.9%)	17/47 (36.2%)

45 surgeons, 12 anesthesiologists, 23 surgical nurses and 10 anesthesiological nurses were working in the surgical area during the study period

**Fig. 1:**
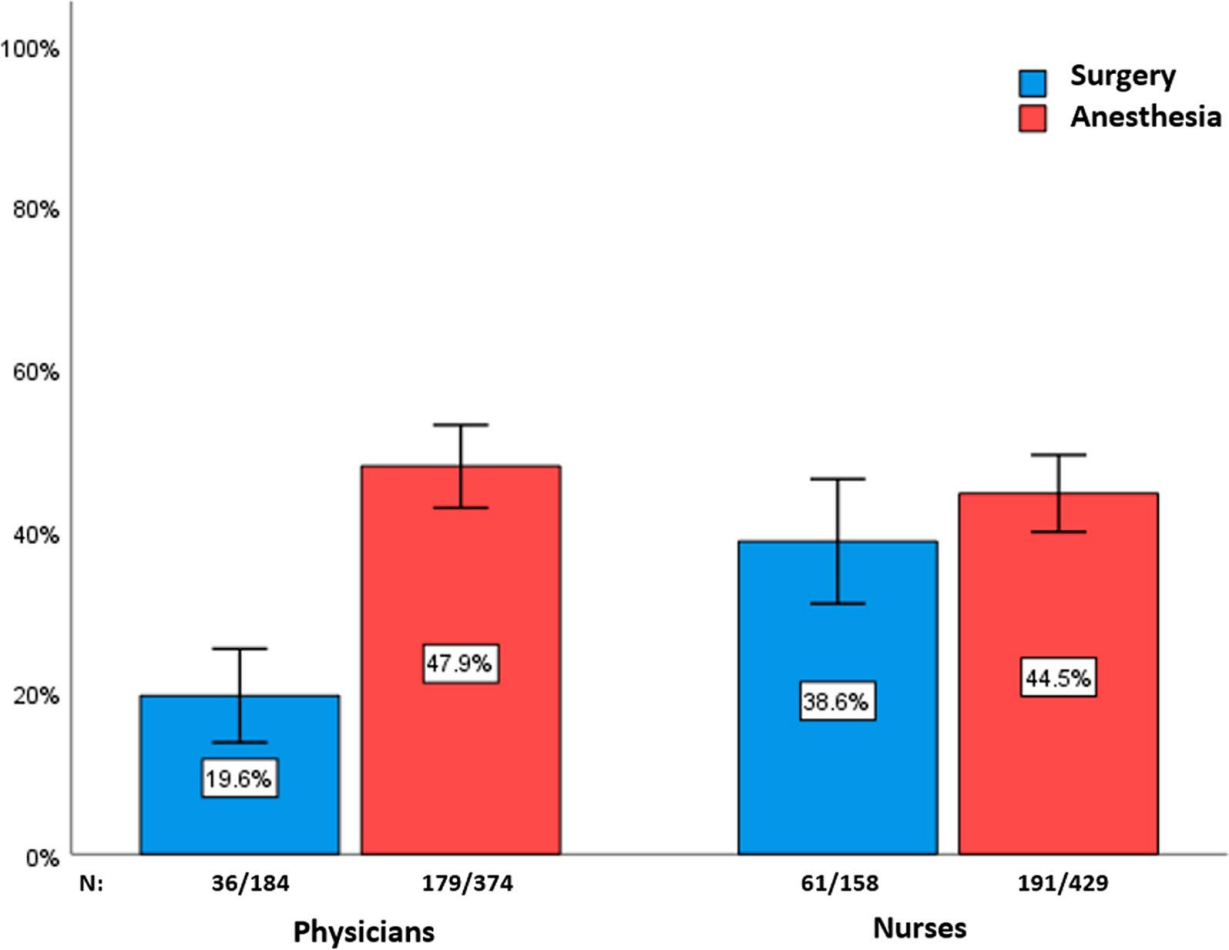
Hygienic hand disinfection compliance by occupational group and medical specialty. Note: 95% confidence intervals are shown

There was a greater difference between surgical and anesthesia staff (28.4% vs. 46.1%, *p* < 0.001) than between physicians and nurses (38.5% vs. 42.9%, *p* = 0.13). Figure 1 shows that there was a significant difference in hand hygiene compliance between surgeons and anesthesiologists (19.6% vs. 47.9%, *p* < 0.001), whereas no equivalent difference pertained to the nursing staff (surgical nurses: 38.6% vs. anesthesia nurses: 44.5%, *p* = 0.199). Correspondingly, the interaction term between occupational group and medical specialty was significant (*p* < 0.001) in the multiple logistic regression analysis for overall compliance. In this model (“TOTAL”-column of Table 2), in which sex, place of observation (inside and outside the operation theatre) and pediatric versus adult surgical context were adjusted for, both nurses and anesthesia staff had higher odds for compliance than physicians and surgery staff, respectively (the crude odds ratios are shown in parentheses).

**Table 2:** Overall hygienic hand disinfection compliance by occupational group and medical specialty: results of three logistic regressions (total, physicians, nurses)

Variables	Total			Physicians			Nurses		
	N (opportunities)	Wald	*p*						
Interaction occupational group × medical specialty	1145	18.6	*p* < 0.001						

	N (opportunities)	OR (crude)	95% CI (crude)	N	OR (crude)	95% CI (crude)	N	OR (crude)	95% CI (crude)
Occupational group									
Physicians	558	Ref							
Nurses	587	**1.7** (1.2)	**1.2–2.4** (0.9–1.5)						
Medical specialty									
Surgery	342	Ref		184	Ref		158	Ref	
Anesthesia	803	**2.0 (2.2)**	**1.5–2.7 (1.6–2.8)**	374	**4.8 (3.8)**	**3.0–7.6 (2.5–5.7)**	429	1.3 (1.3)	0.7–2.3 (0.9–1.9)
Sex									
Men	601	Ref		426	Ref		175	Ref	
Women	544	0.9 (1.2)	0.7–1.2 (1.0–1.5)	132	1.0 **(1.7)**	0.6–1.5 (**1.2–2.5)**	412	0.8 (0.8)	0.5–1.2 (0.6–1.2)
Location									
Inside operation theatre	463	Ref		230	Ref		233	Ref	
Outside operation theatre	682	1.2 (1.2)	0.9–1.7 (0.9–1.5)	328	**1.7** (1.2)	**1.1–2.4** (0.8–1.7)	354	0.9 (1.1)	0.5–1.4 (0.8–1.6)
Operation theatres									
Adults	867	Ref		424	Ref		443	Ref	
Pediatric	278	**1.9 (1.8)**	**1.4–2.6 (1.4–2.3)**	134	**3.0 (2.4)**	**1.9–4.7 (1.6–3.5)**	144	1.4 (1.4)	1.0–2.1 (0.9–2.0)

Significant results (*p* < 0.05) are displayed in bold

*OR* odds ratio, *95% CI* 95% confidence interval, *Ref.* reference

Inspection of regression models stratified either for medical specialty or occupational group showed two results. First, as Additional file 1 shows, in adjusted models occupational group had no significant association with compliance in surgery (OR = 2.8, 95% CI 0.6–13.9) or in anesthesia (OR = 0.8, 95% CI 0.6–1.0). Second, a different pattern emerged for medical specialty. As shown in the columns “PHYSICIANS” and “NURSES” of Table 2, the odds for compliance were higher for anesthesia staff than for surgical staff among physicians (OR = 4.8, 95% CI 3.0–7.6) but not nurses (OR = 1.3, 95% CI 0.7–2.3). This reflects the abovementioned differences between 47.9% for anesthesiologists versus 19.6% for surgeons and 44.5% for anesthesia nurses verus 38.6% for surgical nurses, respectively (see Fig. 1).

In addition, Table 2 shows that the odds for physicians’ overall compliance were significantly higher in the operation theatre for pediatric patients (OR = 3.0, 95% CI 1.9–4.7). Among physicians, compliance was also higher when hand hygiene opportunities occurred outside of the operation theatre (OR = 1.7, 95% CI 1.1–2.4).

The results of the analyses stratified by WHO-5 indications are presented in Additional file 2 and Additional file 3. Mostly, the compliance differences by occupational group and medical specialty in terms of an especially large difference between anesthesia and surgery among physicians pertained to individual indications (per WHO-5) as well. There are two notable exceptions. First, since no opportunities before aseptic tasks at all were observed for the surgeons, no comparison was possible for the variable medical specialty among physicians (Additional file 2, B). Thus, the significantly lower odds for compliance associated with nurses (OR = 0.4, 95% CI 0.2–0.7; see Additional file 3, B) should be seen with caution since nurses from surgery and anesthesia in this case are compared with anesthesiologists only. Second, the interaction between medical specialty and occupational group was not significant in case of opportunities after contact with patient surroundings, even though the difference by medical specialty was considerably higher among physicians (OR = 9.7, 95% CI 2.4–40.0) than nurses (OR = 2.2, 95% CI 0.6–7.9) in numerical terms (see Additional file 3, E).

## Discussion

This study investigated HH compliance (in terms of hygienic hand disinfection as recommended by WHO-5) in the surgical area of a specialized orthopedic hospital. Overall, a compliance of approximately 41% was observed. Thus, while in line with findings from other studies [5, 6]—which sometimes have reported lower compliance levels [12]—HH compliance clearly merits improvement in the present setting. There might be different explanations for this finding. First, the workload in a specialized orthopedic facility with high patient turnover and multitasking (especially for the anesthesia staff) may be relevant, as described before in another setting [9]. Second, lack of time might also be an issue (as observed in intensive care [13]).

While we, like others [1, 2], did observe higher compliance for nurses than for physicians, the differences across the medical specialties involved, i.e., anesthesia versus surgery, were considerably larger. Anesthesia staff had higher compliance than surgical staff, especially among physicians. These patterns are notable, especially since previous studies in the surgery setting have often focused on the hand hygiene performance of anesthesia staff only [7]. One explanation might be that surgeons associate hand hygiene in the surgical area predominantly with surgical hand disinfection immediately prior to the operative procedure and tend to overlook indications for hygienic hand disinfection.

In the study clinic, multimodal surgical site infection-preventive interventions have taken place since 2007 [14]. These included hand hygiene education for nurses and physicians in the wards. Since 2017, anesthesia staff (nurses and physicians) in the surgical area has been trained yearly with respect to hand hygiene. Future interventions to increase hand hygiene should also be directed at surgeons and explicitly address the immediate perioperative setting.

Considering the overall level of HH compliance of approximately 41% in the present study, the impact of the abovementioned interventions remains disillusioning. This underlines that more research on sustainable interventions is urgently needed. Using behavioral change principles for these interventions might be a promising concept but further research is needed as stated in a systematic review by Srigley et al. [15]. In this context, Clancy et al. state in a systemic review of HH related clinical trials that future research should also focus on which intervention types work best for different professional groups [1]. Furthermore, studies should find ways to initiate or maintain collection and evaluation of HH compliance data at regular and long-term follow-up intervals [1].

Looking at different medical specialties, varying hand hygiene compliance (e.g. surgical: 72%, medical: 71%, pediatric: 78%, rehabilitation: 66%) in non-intensive care units have been reported within the reference data of the national hand hygiene campaign in Germany [10].The overall median compliance in the aforementioned study was 73%, which is higher than the observed 41% in our study setting. This again underlines the need to assess and address hand hygiene within an operating area of hospitals. Nonetheless, we are aware that different settings (such as intensive care units, non-intensive care units, and operating theatres) are only comparable to a limited extent due to different conditions, including staffing and workload. Moreover, hand hygiene compliance might be measured differently depending on the respective setting.

In the present study, compliance was higher in the operation theatre for pediatric patients. Healthcare workers in pediatric patient care settings are known to have higher HH compliance [10, 16]. One reason may be that pediatric patient populations rely greatly on hands-on-care [17]. Healthcare workers involved in pediatrics are aware of this and use good HH to protect their vulnerable patients. In fact, surgical site infections following, for instance, pediatric spinal deformity surgery represent a severe complication [18], with possibly consequential lifelong damage starting at a comparatively young age. This might be another reason for the comparatively high HH compliance in this setting.

The study has potential limitations and strengths. It is a single-center study in a highly specialized patient care setting of an orthopedic university clinic. Thus, our findings might not apply to other settings. However, nearly all operative procedures observed were elective, which may imply some transferability to comparable institutions providing elective orthopedic surgery (such as knee and hip arthroplasty). While possibly overestimating compliance through the Hawthorne effect [19], this does not necessarily invalidate specific comparisons between subgroups such as occupational groups or medical specialties. Observation took place in late summer 2020 after the first wave of the coronavirus disease 2019 (COVID-19) pandemic in Germany. On the one hand, this might have influenced the hand hygiene performance in our study, as reported previously by others [20]. On the other hand, in our experience, elective orthopedic surgery was not a clinical hotspot of the COVID-19 pandemic. Moreover, improvements of hand hygiene compliance during the first wave of the COVID-19 pandemic were not always sustained [21]. Thus, the impact may have been not so substantial. Finally, we did not track single observation events (i.e., hand hygiene opportunities) to specific physicians or nurses. Thus, multilevel analysis taking into account the individual level was not viable. However, the observer was instructed to observe as many different healthcare workers as possible in order to reduce bias due to consistent behavior of individual persons. In our study clinic, alcoholic liquid hand-rub solution is used for HH purposes, which is in very widespread use in Germany. Of note, other formulations of alcoholic hand rub solution (such as gels or foams) have been positively evaluated in terms of high acceptance, which may also have affected compliance in our study [22].

Regarding strengths, most importantly, patients were followed continuously, i.e., during their entire stay in the surgical area. Our results reflect a comprehensive and probably realistic approximation of the “true” number of hand hygiene opportunities in this specific setting, with the number of missed opportunities probably in the lower single digit range. Furthermore, the inclusion of surgical staff in a study located within the specific setting of the surgical area itself addresses a relevant stakeholder group whose role in infection prevention has tended to be under-researched and potentially underestimated [23].

In conclusion, this study emphasizes that there is a clear need for improvement of HH compliance in the immediate pre, intra- and postoperative patient care setting in orthopedics. Efforts are particularly needed to increase compliance among surgeons. However, the anesthesia care team also needs to be addressed, as it is responsible for the majority of aseptic tasks. Given the abovementioned previous intervention efforts in the present setting, more strictly tailored interventions (e.g., strategies addressing different occupational groups’ needs empirically assessed beforehand [24, 25]), may be a promising option in orthopedic surgery.

## Supplementary Information

**Additional file 1.** Overall hygienic hand disinfection compliance by occupational group and medical specialty: results of two logistic regressions (surgery, anesthesia).

**Additional file 2.** (**A**–**E**) Hygienic hand disinfection compliance by occupational group and medical specialty, stratified according to WHO-5.

**Additional file 3.** (**A**–**E**) Hygienic hand disinfection compliance by occupational group and medical specialty, stratified according to WHO-5: results of three logistic regressions (total, physicians, nurses).

## Data Availability

The datasets generated and/or analyzed during the current study are available from the corresponding author on reasonable request.

## Abbreviations

HH: Hand hygiene
WHO-5: WHO “My 5 moments for hand hygiene”-model
MHH: Hannover Medical School
95% CI: 95% Confidence interval
OR: Odds ratio
COVID-19: Coronavirus disease 2019

## Declarations

### Further note

The results of this study have partly been presented at the 73rd Annual Conference of the German Society for Hygiene and Microbiology.
