# Supplementary material for: Compliance with hand disinfection in the surgical area of an orthopedic university clinic: results of an observational study
Source: Antimicrob Resist Infect Control. 2022 Jan 31;11:22. doi: 10.1186/s13756-022-01058-2 (PMC8802282; doi:10.1186/s13756-022-01058-2)
Supplement: Supplementary file 1 — Additional file 1. Overall hygienic hand disinfection compliance by occupational group and medical specialty: results of two logistic regressions (surgery, anesthesia). [file 13756_2022_1058_MOESM1_ESM.pdf]

Additional file 1. Overall hygienic hand disinfection compliance by occupational group and medical specialty: results of two logistic regressions (surgery, anesthesia).

**VARIABLES**		**SURGERY**			**ANESTHESIA**		
		**N**	**OR (crude)**	**95%-CI (crude)**	**N**	**OR (crude)**	**95%-CI (crude)**
**Occupational group**	Physicians	184	Ref.		374	Ref.	
	Nurses	158	2.8 **(2.6)**	0.6-13.9 **(1.6-4.2)**	429	0.8 (0.9)	0.6-1.0 (0.7-1.2)
**Sex**	Men	165	Ref.		436	Ref.	
	Women	177	0.5 **(2.1)**	0.1-2.2 **(1.3-3.5)**	367	0.9 (1.1)	0.7-1.3 (0.8-1.4)
**Location**	Inside operation theatre	211	Ref.		252	Ref.	
	Outside operation theatre	131	**0.3 (0.3)**	**0.2-0.7 (0.2-0.5)**	551	**1.6 (1.4)**	**1.1-2.2 (1.1-1.9)**
**Operation theatres**	Adults	262	Ref.		605	Ref.	
	Pediatric	80	**2.2 (1.8)**	**1.2-3.8 (1.0-3.0)**	198	**1.9 (1.8)**	**1.3-2.6 (1.3-2.5)**

Note: OR: odds ratio, 95%-CI: 95% confidence interval, Ref.: reference; significant results (p<0.05) are displayed in bold.

Additional file to: Claas Baier, Maren Tinne, Thomas von Lengerke, Frank Gossé, Ella Ebadi. Compliance with hand disinfection in the surgical area of an orthopedic university clinic: results of an observational study. DOI: 10.1186/s13756-022-01058-2
