# Supplementary material for: Compliance with hand disinfection in the surgical area of an orthopedic university clinic: results of an observational study
Source: Antimicrob Resist Infect Control. 2022 Jan 31;11:22. doi: 10.1186/s13756-022-01058-2 (PMC8802282; doi:10.1186/s13756-022-01058-2)
Supplement: Supplementary file 2 — Additional file 2. (A–E) Hygienic hand disinfection compliance by occupational group and medical specialty, stratified according to WHO-5. [file 13756_2022_1058_MOESM2_ESM.pdf]

Additional file 2. A-E: Hygienic hand disinfection compliance by occupational group and medical specialty, stratified according to WHO-5.

A. Before patient contact (n=190)

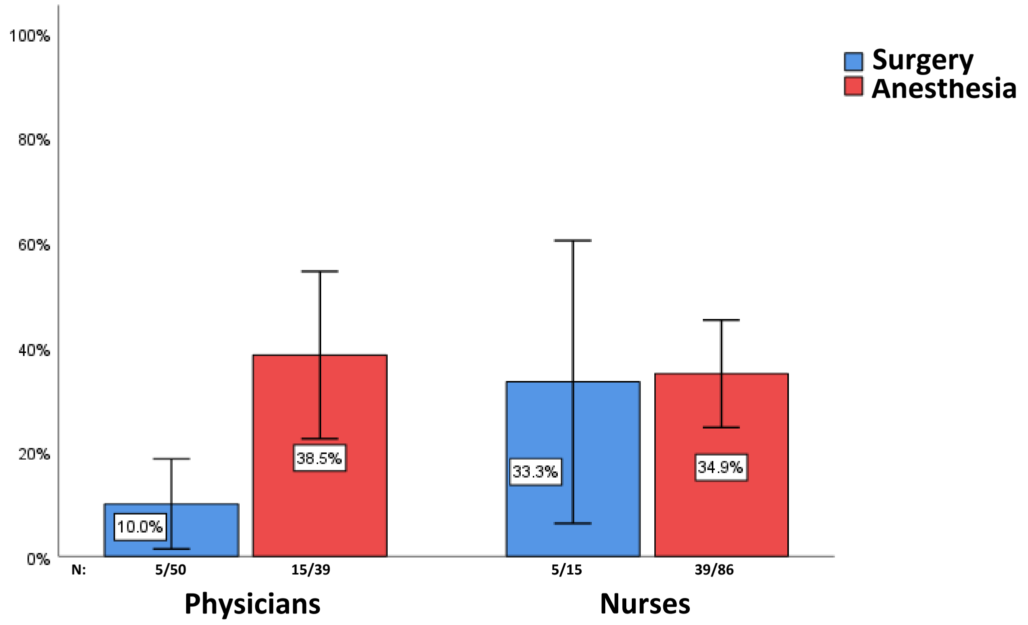

Note: 95% confidence intervals are shown.

Additional file to: Claas Baier, Maren Tinne, Thomas von Lengerke, Frank Gossé, Ella Ebadi. Compliance with hand disinfection in the surgical area of an orthopedic university clinic: results of an observational study. DOI: 10.1186/s13756-022-01058-2

B. Before aseptic task (n=277)

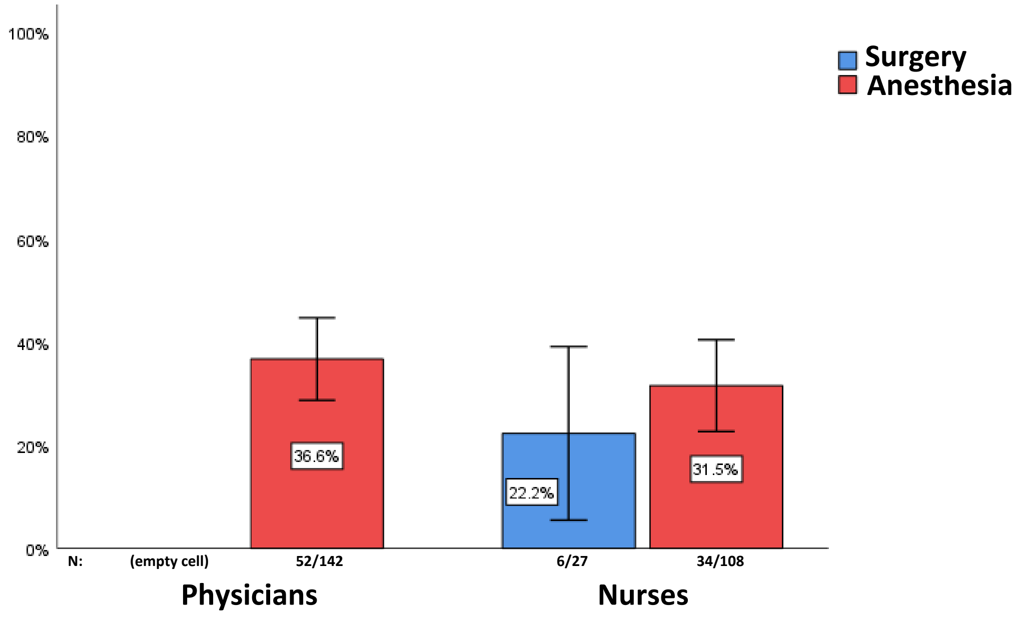

Note: 95% confidence intervals are shown.

Additional file to: Claas Baier, Maren Tinne, Thomas von Lengerke, Frank Gossé, Ella Ebadi. Compliance with hand disinfection in the surgical area of an orthopedic university clinic: results of an observational study. DOI: 10.1186/s13756-022-01058-2

C. After body fluid exposure (n=198)

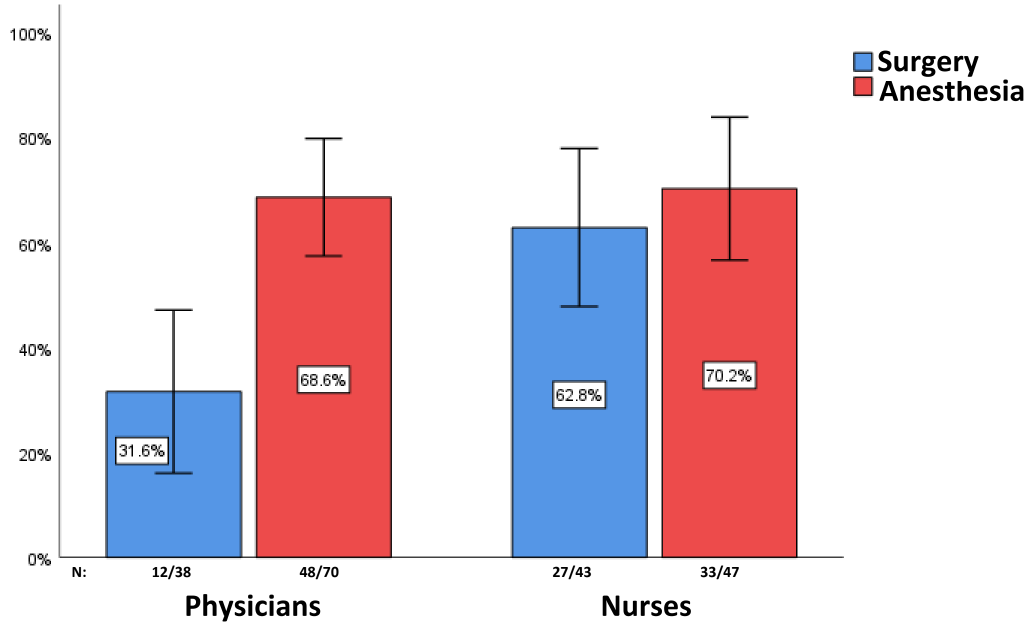

Note: 95% confidence intervals are shown.

Additional file to: Claas Baier, Maren Tinne, Thomas von Lengerke, Frank Gossé, Ella Ebadi. Compliance with hand disinfection in the surgical area of an orthopedic university clinic: results of an observational study. DOI: 10.1186/s13756-022-01058-2

D. <u>After patient contact</u> (n=221)

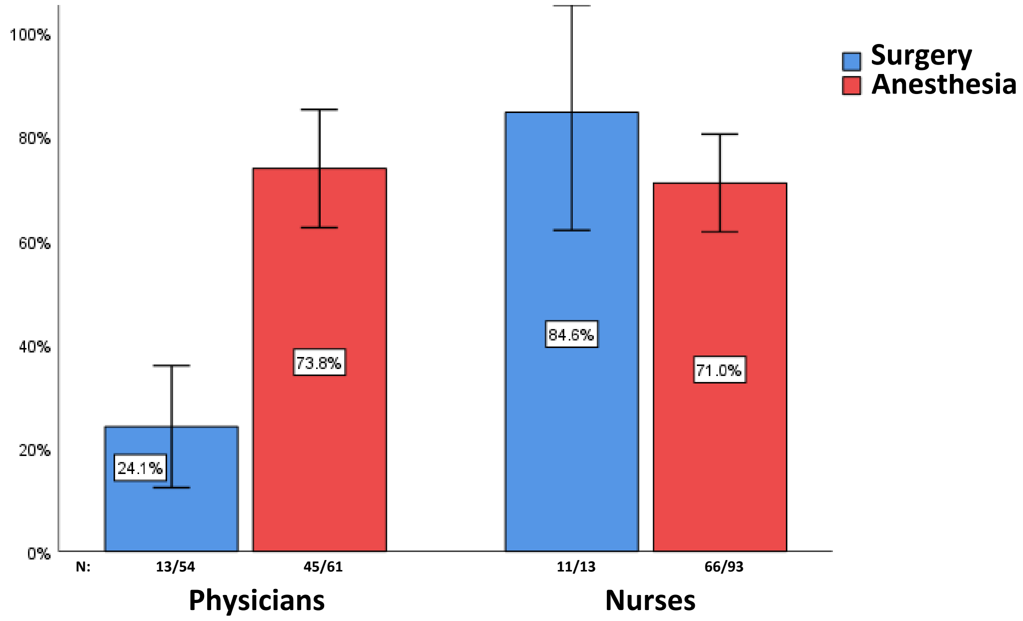

Note: 95% confidence intervals are shown.

Additional file to: Claas Baier, Maren Tinne, Thomas von Lengerke, Frank Gossé, Ella Ebadi. Compliance with hand disinfection in the surgical area of an orthopedic university clinic: results of an observational study. DOI: 10.1186/s13756-022-01058-2

E. After contact with patient surroundings (n=259)

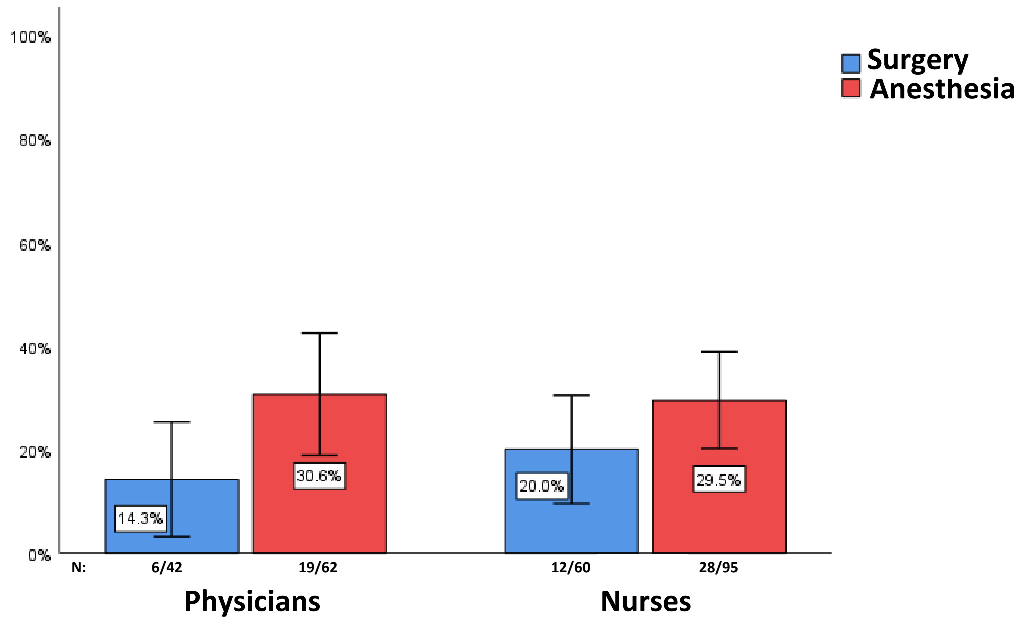

Note: 95% confidence intervals are shown.

Additional file to: Claas Baier, Maren Tinne, Thomas von Lengerke, Frank Gossé, Ella Ebadi. Compliance with hand disinfection in the surgical area of an orthopedic university clinic: results of an observational study. DOI: 10.1186/s13756-022-01058-2
