# Supplementary material for: Compliance with hand disinfection in the surgical area of an orthopedic university clinic: results of an observational study
Source: Antimicrob Resist Infect Control. 2022 Jan 31;11:22. doi: 10.1186/s13756-022-01058-2 (PMC8802282; doi:10.1186/s13756-022-01058-2)
Supplement: Supplementary file 3 — Additional file 3. (A–E) Hygienic hand disinfection compliance by occupational group and medical specialty, stratified according to WHO-5: results of three logistic regressions (total, physicians, nurses). [file 13756_2022_1058_MOESM3_ESM.pdf]

Additional file 3. A-E: Hygienic hand disinfection compliance by occupational group and medical specialty, stratified according to WHO-5: results of three logistic regressions (total, physicians, nurses).

A. Before patient contact (n=190)

**VARIABLES**		**TOTAL**			**PHYSICIANS**			**NURSES**		
		**N** (opportunities)	**Wald**	**p**						
**Interaction Occupational Group x Medical Specialty**		190	**6.39**	**p = 0.012**						
		**N** (opportunities)	**OR (crude)**	**95%-CI (crude)**	**N**	**OR (crude)**	**95%-CI (crude)**	**N**	**OR (crude)**	**95%-CI (crude)**
**Occupational group**	Physicians	89	Ref.							
	Nurses	101	**2.9** (1.8)	**1.1-7.7** (1.0-3.5)						
**Medical specialty**	Surgery	65	Ref.		50	Ref.		15	Ref.	
	Anesthesia	125	2.1 **(3.1)**	0.9-5.0 **(1.4-6.7)**	39	**5.9 (5.6)**	**1.8-19.3 (1.8-17.4)**	86	0.4 (1.1)	0.1-1.9 (0.3-3.4)
**Sex**	Men	114	Ref.		74	Ref.		40	Ref.	
	Women	76	0.7 (1.0)	0.3-1.5 (0.5-1.9)	15	0.8 (1.3)	0.2-3.4 (0.4-4.7)	61	0.7 (0.6)	0.3-1.8 (0.2-1.3)
**Location**	Inside operation theatre	54	Ref.		21	Ref.		33	Ref.	
	Outside operation theatre	136	1.7 (1.2)	0.8-4.0 (0.6-2.5)	68	0.9 (0.6)	0.3-3.2 (0.2-2.0)	68	3.3 (2.1)	1.0-11.5 (0.8-5.2)
**Operation theatres**	Adults	145	Ref.		67	Ref.		78	Ref.	
	Pediatric	45	0.4 (0.5)	0.2-1.1 (0.2-1.0)	22	1.0 (1.0)	0.3-3.7 (0.3-3.2)	23	**0.2 (0.2)**	**0.1-0.7 (0.1-0.8)**

Note: OR: odds ratio, 95%-CI: 95% confidence interval, Ref.: reference; significant results (p<0.05) are displayed in bold.

Additional file to: Claas Baier, Maren Tinne, Thomas von Lengerke, Frank Gossé, Ella Ebadi. Compliance with hand disinfection in the surgical area of an orthopedic university clinic: results of an observational study. DOI: 10.1186/s13756-022-01058-2

B. Before aseptic task (n=277)*

VARIABLES		TOTAL		
		**N** (opportunities)	**OR (crude)**	**95%-CI (crude)**
**Occupational group**	Physicians	142	Ref.	
	Nurses	135	**0.4** (0.7)	**0.2-0.7** (0.4-1.2)
**Medical specialty**	Surgery	27	Ref.	
	Anesthesia	250	0.4 (1.8)	0.1-1.4 (0.7-4.7)
**Sex**	Men	144	Ref.	
	Women	133	1.2 (1.1)	0.6-2.3 (0.7-1.9)
**Location**	Inside operation theatre	117	Ref.	
	Outside operation theatre	160	**4.2 (2.5)**	**2.1-8.5 (1.4-4.2)**
**Operation theatres**	Adults	217	Ref.	
	Pediatric	60	**2.1 (2.1)**	**1.1-4.2 (1.2-3.8)**

Note: OR: odds ratio, 95%-CI: 95% confidence interval, Ref.: reference; significant results ($p<0.05$) are displayed in bold. *No separate logistic regressions for the subgroups nurses and physicians due to no observed hand hygiene opportunities before aseptic procedures among surgeons.

C. After body fluid exposure (n=198)

VARIABLES		TOTAL			PHYSICIANS			NURSES		
		**N** (opportunities)	**Wald**	**p**						
**Interaction Occupational Group x Medical Specialty**		198	**5.55**	**p = 0.018**						
		**N** (opportunities)	**OR (crude)**	**95%-CI (crude)**	**N**	**OR (crude)**	**95%-CI (crude)**	**N**	**OR (crude)**	**95%-CI (crude)**
**Occupational group**	Physicians	108	Ref.							
	Nurses	90	**2.6** (1.6)	**1.1-6.1** (0.9-2.9)						
**Medical specialty**	Surgery	81	Ref.		38	Ref.		43	Ref.	
	Anesthesia	117	2.5 **(2.4)**	0.9-6.9 **(1.3-4.4)**	70	**7.2 (4.7)**	**2.0-25.6 (2.0-11.1)**	47	1.3 (1.4)	0.1-16.6 (0.6-3.4)
**Sex**	Men	101	Ref.		83	Ref.		18	Ref.	
	Women	97	0.8 (1.6)	0.3-1.8 (0.9-2.8)	25	0.5 (1.6)	0.2-1.6 (0.6-4.0)	72	1.2 (1.0)	0.3-4.3 (0.3-3.0)
**Location**	Inside operation theatre	105	Ref.		59	Ref.		46	Ref.	
	Outside operation theatre	93	1.0 **(1.9)**	0.4-2.7 **(1.1-3.5)**	49	1.0 **(2.4)**	0.3-3.0 **(1.1-5.4)**	44	1.0 (1.4)	0.1-12.7 (0.6-3.4)
**Operation theatres**	Adults	150	Ref.		81	Ref.		69	Ref.	
	Pediatric	48	**3.1 (2.4)**	**1.3 -7.0 (1.1-4.9)**	27	**5.3 (3.0)**	**1.7-16.8 (1.1-7.7)**	21	1.7 (1.8)	0.5-5.4 (0.6-5.6)

Note: OR: odds ratio, 95%-CI: 95% confidence interval, Ref.: reference; significant results (p<0.05) are displayed in bold.

Additional file to: Claas Baier, Maren Tinne, Thomas von Lengerke, Frank Gossé, Ella Ebadi. Compliance with hand disinfection in the surgical area of an orthopedic university clinic: results of an observational study. DOI: 10.1186/s13756-022-01058-2

D. After patient contact (n=221)

**VARIABLES**		**TOTAL**			**PHYSICIANS**			**NURSES**		
		**N** (opportunities)	**Wald**	**p**						
**Interaction Occupational Group x Medical Specialty**		221	**5.21**	**p = 0.022**						
		**N** (opportunities)	**OR (crude)**	**95%-CI (crude)**	**N**	**OR (crude)**	**95%-CI (crude)**	**N**	**OR (crude)**	**95%-CI (crude)**
**Occupational group**	Physicians	115	Ref.							
	Nurses	106	2.8 **(2.6)**	1.0-8.2 **(1.5-4.6)**						
**Medical specialty**	Surgery	67	Ref.		54	Ref.		13	Ref.	
	Anesthesia	154	**2.9 (4.6)**	**1.1-7.5 (2.5.-8.5)**	61	**8.7 (8.9)**	**3.3-23.0 (3.8-20.7)**	93	0.8 (0.4)	0.1-4.9 (0.1-2.1)
**Sex**	Men	129	Ref.		90	Ref.		39	Ref.	
	Women	92	0.8 **(2.2)**	0.4-1.8 **(1.2-3.9)**	25	1.1 **(3.2)**	0.3-3.7 **(1.2-8.5)**	67	0.7 (0.9)	0.3-1.8 (0.4-2.1)
**Location**	Inside operation theatre	71	Ref.		29	Ref.		42	Ref.	
	Outside operation theatre	150	**0.3 (0.3)**	**0.1-0.7 (0.1-0.5)**	86	**0.3 (0.2)**	**0.1-0.9 (0.1-0.6)**	64	0.3 (0.4)	0.1-1.0 (0.1-1.0)
**Operation theatres**	Adults	161	Ref.		85	Ref.		76	Ref.	
	Pediatric	60	**3.2 (2.1)**	**1.5 -7.1 (1.1-4.1)**	30	**3.3** 2.0	**1.1-10.2** (0.9-4.8)	30	3.0 (2.3)	1.0-9.3 (0.8-6.8)

Note: OR: odds ratio, 95%-CI: 95% confidence interval, Ref.: reference; significant results (p<0.05) are displayed in bold.

Additional file to: Claas Baier, Maren Tinne, Thomas von Lengerke, Frank Gossé, Ella Ebadi. Compliance with hand disinfection in the surgical area of an orthopedic university clinic: results of an observational study. DOI: 10.1186/s13756-022-01058-2

E. After contact with patient surroundings (n=259)

VARIABLES		TOTAL			PHYSICIANS			NURSES		
		**N** (opportunities)	**Wald**	**p**						
**Interaction Occupational Group x Medical Specialty**		259	1.93	p = 0.165						
		**N** (opportunities)	**OR (crude)**	**95%-CI (crude)**	**N**	**OR (crude)**	**95%-CI (crude)**	**N**	**OR (crude)**	**95%-CI (crude)**
**Occupational group**	Physicians	104	Ref.							
	Nurses	155	1.5 (1.1)	0.7-3.2 (0.6-2.0)						
**Medical specialty**	Surgery	102	Ref.		42	Ref.		60	Ref.	
	Anesthesia	157	**2.1 (2.0)**	**1.1-4.2 (1.1.-3.7)**	62	**9.7** (2.7)	**2.4-40.0** (1.0-7.3)	95	2.2 (1.7)	0.6-7.9 (0.8-3.6)
**Sex**	Men	113	Ref.		76	Ref.		37	Ref.	
	Women	146	0.9 (1.0)	0.4-1.8 (0.6-1.8)	28	1.9 (1.8)	0.6-6.4 (0.7-4.7)	118	0.7 (0.6)	0.3-1.7 (0.3-1.5)
**Location**	Inside operation theatre	116	Ref.		40	Ref.		76	Ref.	
	Outside operation theatre	143	1.7 (1.3)	0.7-3.9 (0.7-2.3)	64	**7.0** (1.4)	**1.7-28.5** (0.6-3.8)	79	0.7 (1.2)	0.2-1.9 (0.6-2.6)
**Operation theatres**	Adults	194	Ref.		77	Ref.		117	Ref.	
	Pediatric	65	**3.3 (2.9)**	**1.7-6.2 (1.6-5.4)**	27	**8.2 (5.0)**	**2.5-26.8 (1.9-13.3)**	38	2.3 (2.0)	1.0-5.3 (0.9-4.5)

Note: OR: odds ratio, 95%-CI: 95% confidence interval, Ref.: reference; significant results ($p<0.05$) are displayed in bold.

Additional file to: Claas Baier, Maren Tinne, Thomas von Lengerke, Frank Gossé, Ella Ebadi. Compliance with hand disinfection in the surgical area of an orthopedic university clinic: results of an observational study. DOI: 10.1186/s13756-022-01058-2
